# Inoculation With Indigenous Rhizosphere Microbes Enhances Aboveground Accumulation of Lead in *Salix integra* Thunb. by Improving Transport Coefficients

**DOI:** 10.3389/fmicb.2021.686812

**Published:** 2021-08-04

**Authors:** Xiao-yun Niu, Shao-kun Wang, Jian Zhou, Dong-liu Di, Pai Sun, Da-zhuang Huang

**Affiliations:** College of Landscape Architecture and Tourism, Hebei Agricultural University, Baoding, China

**Keywords:** indigenous rhizosphere microbes, co-inoculation, transfer coefficient, widely targeted metabolome analysis, heavy metal bioavailability, plant-microbial remediation

## Abstract

The application of plant–microbial remediation of heavy metals is restricted by the difficulty of exogenous microbes to form large populations and maintain their long-term remediation efficiency. We therefore investigated the effects of inoculation with indigenous heavy-metal-tolerant rhizosphere microbes on phytoremediation of lead (Pb) by *Salix integra.* We measured plant physiological indexes and soil Pb bioavailability and conducted widespread targeted metabolome analysis of strains to better understand the mechanisms of enhance Pb accumulation. Growth of *Salix integra* was improved by both single and co-inoculation treatments with *Bacillus* sp. and *Aspergillus niger*, increasing by 14% in co-inoculated plants. Transfer coefficients for Pb, indicating mobility from soil via roots into branches or leaves, were higher following microbial inoculation, showing a more than 100% increase in the co-inoculation treatment over untreated plants. However, Pb accumulation was only enhanced by single inoculation treatments with either *Bacillus* sp. or *Aspergillus niger*, being 10% greater in plants inoculated with *Bacillus* sp. compared with uninoculated controls. Inoculation mainly promoted accumulation of Pb in aboveground plant parts. Superoxide dismutase and catalase enzyme activities as well as the proline content of inoculated plants were enhanced by most treatments. However, soil urease and catalase activities were lower in inoculated plants than controls. Proportions of acid-soluble Pb were 0.34 and 0.41% higher in rhizosphere and bulk soil, respectively, of plants inoculated with *Bacillus* sp. than in that of uninoculated plants. We identified 410 metabolites from the microbial inoculations, of which more than 50% contributed to heavy metal bioavailability; organic acids, amino acids, and carbohydrates formed the three major metabolite categories. These results suggest that both indigenous *Bacillus* sp. and *Aspergillus niger* could be used to assist phytoremediation by enhancing antioxidant defenses of *Salix integra* and altering Pb bioavailability. We speculate that microbial strains colonized the soil and plants at the same time, with variations in their metabolite profiles reflecting different living conditions. We also need to consider interactions between inocula and the whole microbial community when applying microbial inoculation to promote phytoremediation.

## Introduction

Heavy-metal contamination of soil is an increasingly urgent global problem causing decreased plant growth and threatening human and animal health (Rahman et al., 2017; O’Connor et al., 2018; Tian et al., 2018). Pb is a widespread heavy metal in soil, and its accumulation causes severe harm to humans, particularly children. Many strategies have been developed to reduce the effects of Pb on ecosystems and humans, although most have high costs and can cause secondary pollution (Niu et al., 2019). Phytoremediation offers a low-cost approach without secondary contamination; however, its lower remediation efficiency hinders its large-scale application (Ye et al., 2019). Researchers have therefore explored possible methods of increasing remediation efficiency, including effective agronomic practices (Hamid et al., 2018, 2019; Leite and Monteiro, 2019; Wu et al., 2020c), genetic engineering technology (Zhang et al., 2014; Feng et al., 2018), and inoculation with microbes (Jian et al., 2019; Tao et al., 2020; Wu et al., 2020b). Among these technologies, we have focused on plant–microbial remediation due to its low cost and high efficiency for enhancing phytoremediation (Wang et al., 2020).

Microbes such as plant-growth-promoting bacteria (Guo et al., 2020; Wu et al., 2020a), endophytes (Santoyo et al., 2016; Wood et al., 2016; Ma et al., 2017; Wang et al., 2020), and ectomycorrhiza (Coninx et al., 2017; Szuba et al., 2017) can increase the phytoremediation of heavy metals in contaminated soil (Ma et al., 2016; Santoyo et al., 2016; Wood et al., 2016). Possible mechanisms include stimulation of plant growth through biosynthesis of phytohormones and increasing plant chlorophyll content (Babu et al., 2015; Gang et al., 2018), promoting the nutrient cycle through production of siderophores, nitrogen fixation, and solubilization of phosphorus (Ma et al., 2017; Lin et al., 2018; Wang L. et al., 2018; Wang Q. et al., 2018), and promoting heavy-metal tolerance and the expression of some metal-transporter genes in inoculated plants (Chen et al., 2014; Pan et al., 2016, 2017). The effects of co-inoculation with different microorganisms have been examined in recent years (Kumar et al., 2017; Haro et al., 2018; Jian et al., 2019). For example, co-inoculation with bacteria able to solubilize phosphorus biosynthesize indole-3-acetic acid, and produce siderophores significantly increases Cu extraction compared with single inoculation in maize (Rojas-Tapias et al., 2014), and co-inoculation with *Sinorhizobium meliloti* and *Agrobacterium tumefaciens* enhances metal phytoextraction by increasing plant growth and antioxidant activities under Cu/Zn stress (Jian et al., 2019). However, the effects of co-inoculation on phytoremediation are very complicated (Tsimilli-Michael et al., 2000; Yu et al., 2005; Aghababaei et al., 2014; Mahohi and Raiesi, 2019).

Proliferation of exogenous microbes inoculated on plants is affected by soil environmental factors, such as nutrients, pH, temperature, and energy sources (Csutak et al., 2010; Shen et al., 2017). Therefore, it is difficult for exogenous microorganisms to form large populations and maintain their long-term remediation efficiency (Igalavithana et al., 2017; Li X.Q. et al., 2017; Li Y. et al., 2017). By contrast, heavy-metal-tolerant indigenous rhizosphere microbes are better adapted to the environment. The mechanism of promotion phytoremediation after inoculation remains unclear, and has been explored indirectly in most previous studies (Tao et al., 2020; Wang et al., 2020). Microbes usually affect phytoremediation through their extracellular metabolites. However, only limited information is available on microbial metabolites, and these should therefore be measured to better understand the mechanisms of promoting phytoremediation.

*Salix integra* Thunb. is a fast-growing woody species, producing a large amount of biomass and a deep root system (Kuzovkina and Volk, 2009; Niu et al., 2019). It is a well-known co-accumulator of Pb/Zn/Cd and is native to China (Liu et al., 2011). There have been several studies on the ability of *Salix integra* to absorb heavy metals (Pb, Cu, Cd, and Zn) (Yang et al., 2014, 2018; Shi et al., 2017; Cao et al., 2018). However, how microbes affect remediation by *Salix Integra* is poorly understood. In this study, we inoculated *Salix integra* with single or mixed indigenous microbes isolated from the rhizosphere soil of *Salix integra* planted in Pb-polluted soil. Our objectives were to identify the indigenous microbes that can improve the uptake efficiency of Pb by *Salix integra*; to study the effects of co-inoculation on Pb uptake by *Salix integra*; and to explore microbial metabolites produced during microbe-assisted phytoremediation to better understand the mechanisms promoting phytoremediation. Our results provide insight into how inoculation with indigenous microbes influences the phytoremediation process.

## Materials and Methods

### Plant Material and Soil Preparation

The study was conducted from March 22 to July 30, 2020, in the greenhouse of the College of Landscape Architecture and Tourism, Hebei Agricultural University (38° 49′ 35″ N, 115° 27′ 7″ E), with a temperature of 25–30°C and natural light conditions. Contaminated soil (a typical meadow cinnamon soil) with an initial Pb concentration of 1,225 mg⋅kg^–1^ was collected from the experimental station at Hebei Agricultural University (38°45′21″ N, 115°24′37″ E). Each pot was filled with 4.3 kg of mixed experimental medium composed of 33.0% sand and 67% contaminated soil by volume. Soil properties were determined using standard analytical procedures (Bao, 2000), and initial soil properties were as follows: pH 7.75; soil organic matter content, 2.03 g⋅kg^–1^; cation exchange capacity (CEC), 8.74 cmol⋅kg^–1^; total nitrogen content, 238 mg⋅kg^–1^; available nitrogen content, 20.18 mg⋅kg^–1^; total phosphorus content, 487 mg⋅kg^–1^; available phosphorus content, 10.14 mg⋅kg^–1^; total potassium content, 7.98 g⋅kg^–1^; available potassium content, 268.4 mg⋅kg^–1^. The soil was sterilized to kill all microbes before use. One-year-old *Salix integra* branches were collected from trees planted at the experimental station at Hebei Agricultural University. Healthy and uniform cuttings were selected and inserted directly into pots on March 22.

### Inoculation Strains and Experimental Design

All strains used for inoculation in this study were isolated from rhizosphere soil of *Salix integra* growing in Pb-contaminated soil at our experimental station at Hebei Agricultural University by screening on medium containing PbNO_3_ (Table 1). Pb tolerance was high among the isolated strains, particularly for *Aspergillus niger*, which could grow on medium containing 4,000 mg Pb^2+^ kg^–1^. The strains were identified using 16S rRNA gene sequence or ITS rDNA analysis for bacteria and fungi, respectively. Universal bacterial primers 27F (AGAGTTTGATCCTGGCTCAG) and 1492R (CGGTTACCTTGTTACGACTT) were used to amplify 16S rRNA gene fragments, and fungal primers ITS1 (TCCGTAGGTGAACCTGCGG) and ITS4 (TCCTCCGCTTATTGATATGC) were used to amplify ITS rDNA fragments. Sequences were used for BLAST search against the NCBI GenBank database (Table 1). It should be noted that although the three strains were isolated from rhizosphere soil, they had also been identified as endophytes in previous research (Aziz et al., 2021; Gimeno et al., 2021).

**TABLE 1:** Blast results of the inoculation strains in this study.

Strain number	Similar strain (Genbank)	Accession number	Similarity (%)	P^2+^ tolerance
Bacterium No. 6	*Bacillus* sp.	MG470715.1	100	600 mg⋅kg^–1^
Fungus No. 2	*Clonostachys rosea*	KX058045.1	99.29	2,100 mg⋅kg^–1^
Fungus No. 3	*Aspergillus niger*	KT192262.1	99.67	4,000 mg⋅kg^–1^

Fungal and bacterial strains were cultured on solid PAD and in liquid LB media, respectively. Bacteria cultures were first centrifuged to remove medium at a speed of 8,000 r⋅min^−1^ and then washed with sterile water three times. Inocula were then prepared by resuspending cells in sterilized saline solution (0.85%; w/v) to achieve an inoculum density of 1 × 10^7^ CFU⋅mL^–1^ (Zhang et al., 2015). Fungi inocula consisted of spores collected from solid medium in a suspension diluted to 1 × 10^7^ CFU⋅mL^–1^ using sterilized saline solution (0.85%). Experiments were conducted with five inoculation treatments as follows: (i) uninoculated control; (ii) inoculation with *Bacillus* sp.; (iii) inoculation with *Clonostachys rosea*; (iv) inoculation with *Aspergillus niger*; and (v) co-inoculation with *Bacillus* sp. and *Aspergillus niger* (1:1, V/V). Each treatment included 15 repeats (pots), which were placed randomly in the greenhouse and repositioned occasionally during the 130 days experimental period. Cuttings showed mature roots after 40 days of growth and were inoculated with microbial strains for the first time. Treatments were applied by evenly spraying and slowly drenching the soil surface of each plant with 40 mL microbial suspension (or control). The same agronomic management measures were applied to all treatments during the study period.

### *Salix interga* Physiological Index for Plant Stress Tolerance

After 60 d of inoculation, mature leaves from each treatment were sampled in four replicates on June 30, 2020. Samples were stored at 4°C, and analyzed within 1 week. Catalase (CAT), superoxide dismutase (SOD), proline (Pro), malondialdehyde (MDA), and soluble protein (Sprotein) were analyzed. CAT activity was estimated following the titration method and expressed in mL KMnO_4_ (g dried soil) ^–1^ (20 min)^–1^ (Li et al., 2009). SOD activity was assayed by monitoring the inhibition of nitroblue tetrazolium (NBT) photochemical reduction (Singh et al., 2020). One unit of SOD activity was defined as the amount required to inhibit 50% the reduction of NBT under light, measured at 560 nm (U⋅h⋅g^–1^). Pro content was measured using an acid ninhydrin solution (Saradhi Alia, 1991). The optical density (OD) of the upper toluene phase was measured at 520 nm. The method described by Singh et al. (2020) was followed to determine MDA content. Lipid peroxidation was expressed as the MDA content in μmol⋅g^–1^. Soluble protein content was determined using the Coomassie brilliant blue staining method, with absorbance determined at 595 nm using a spectrophotometer (Xin et al., 2020).

### Soil Antioxidant Enzyme Activity

After 60 d of inoculation, bulk soils were sampled in four replicates for each treatment on June 30, 2020. Bulk soils were acquired using soil-drilling at a depth of 20–30 cm, with five random samples mixed into one replicate for each pot. Soil materials were preserved at 4°C. After sieving and removing impurities, soil urease (S-UE) and soil catalase (S-CAT) activities in the bulk soils were analyzed within 1 week. Urease activity was determined following a colorimetric method using a spectrophotometer (AA-680, Shimadzu, Kyoto, Japan) at 578 nm (Hu et al., 2014). Catalase assay was the same as that in section “*Salix interga* Physiological Index for Plant Stress Tolerance” for leaves.

### Plant Growth and Total Pb Enrichment

After 90 days of inoculation, plants were harvested and washed carefully with deionized water and then divided into roots, shoots, and leaves. Roots, shoots, and leaves of each sample were oven-dried at 80°C for 24 h, and then dry biomass was measured. Roots, shoots, and leaves were ground and passed through a 60-mesh sieve to measure total Pb content following the method described by Niu et al. (2020).

### Rhizosphere and Bulk Soil Heavy Metal Speciation

After 90 days of inoculation, roots were completely dug out of the pots and shaken gently. Soil adhering to the roots was collected as the rhizosphere soil using a brush. Soil from three plant roots was mixed to create a rhizospheric soil sample. Bulk soils were collected through soil-drilling at a depth of 20–30 cm, and soils from five random samples were combined into one replicate. Rhizospheric and bulk soils were sampled from the four replicates of each treatment. Total Pb was measured following the methods described in section “Plant Growth and Total Pb Enrichment.”

Pb partitioning was assayed following the sequential extraction procedure described by Rauret et al. (1999) using 0.5 g of soil and comprised acid-soluble, reducible, oxidizable, and residual fractions. Acetic acid (0.11 mol⋅L^–1^, 16 h), hydroxylamine hydrochloride (0.5 mol⋅L^–1^, pH 1.5, 16 h), and H_2_O_2_ (8.8 mol⋅L^–1^, 2 × 1 h, 85°C) were first employed for acid-soluble, reducible, and oxidizable extraction, respectively, and this was followed by ammonium acetate extraction (1.0 mol⋅L^–1^). Three acids (4 mL HCl, 2 mL HNO_3_, and 2 mL hydrofluoric acid) were used to digest the residual fraction. The contents of different Pb species were assayed using atomic absorption spectrophotometry.

### Widely Targeted Microbial Metabolome Analysis

#### Sample Preparation and Metabolite Extraction

**FIGURE 1:**
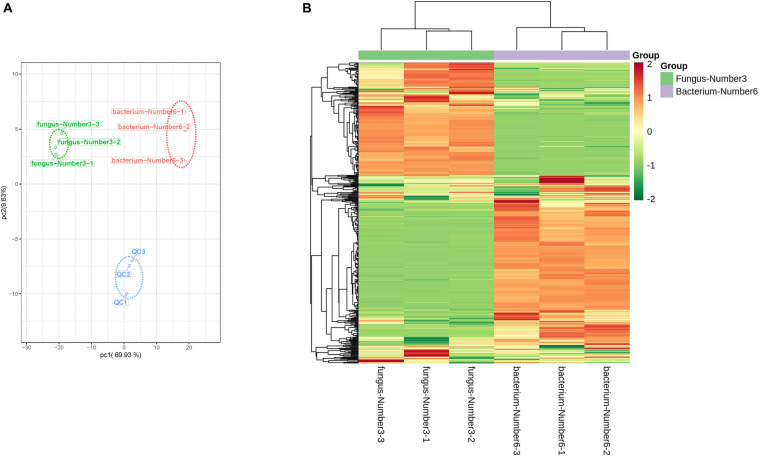
Differences in the metabolites between *Bacillus* sp. and *Aspergillus niger*. **(A)** PCA analysis of the metabolites identified from f *Aspergillus niger* and *Bacillus* sp. Equal volumes of *Aspergillus niger* and *Bacillus* sp. samples were mixed for quality control (QC). **(B)** Cluster analysis of metabolites from samples of *Aspergillus niger* and *Bacillus* sp. The color indicates the level of accumulation of each metabolite from low (green) to high (red). The Z-score represents the deviation from the mean in standard deviation units.

*Aspergillus niger* and *Bacillus* sp. had the largest promoting effects on Pb accumulation. Widely targeted liquid chromatography tandem mass spectrometry (LC-MS/MS)-based metabolite profiling of *Aspergillus niger* and *Bacillus* sp. was therefore performed. *Aspergillus niger* and *Bacillus* sp. were initially cultured in PDA and LB liquid media, respectively, at 37°C. When the OD_600_ reached 0.6 (microbiological logarithmic stage), 100 μL of microbial suspension was added to the fermentation medium as a seed broth and cultured under darkness at pH 8.0, 25°C, and with 350 mg Pb^2+^ L^–1^ (equal to the total content of acid-soluble and reducible Pb^2+^ in the experimental soil) in a 250 mL triangular flask. After 48 h of culturing, microbial suspensions were centrifuged at 1 2,000 r⋅min^–1^ for 10 min at 4°C, and the supernatant was then collected for measuring microbial extracellular metabolism. *Aspergillus niger* and *Bacillus* sp. were both cultured in 12 triangular flasks. Supernatants from four triangular flasks were mixed into one replicate; therefore, three replicates were formed for both *Aspergillus niger* and *Bacillus* sp. Quality control (QC) samples were prepared by mixing equal volumes of the supernatants of *Aspergillus niger* and *Bacillus* sp. to enable the reproducibility of the mass spectrometric results to be assessed; and results are shown in Figure 1A. Supernatants were stored in 50 mL centrifuge tubes under liquid nitrogen before measurement.

#### ESI-Q TRAP-MS/MS Analysis

Each supernatant was defrosted, added to 1 mL of precooled extract (70% methanol solution with 1 μg⋅mL^–1^ of 2 chlorophenylalanine as the internal standard), and vortexed for 1 min. The extracts were then quick-frozen for 3 min in liquid nitrogen, defrosted for 3 min on ice, and then vortexed for 2 min. This process was repeated three times, and the supernatants were then centrifuged at 12,000 r⋅min^–1^ for 10 min at 4°C. Supernatants were analyzed using an LC-ESI-MS/MS system (HPLC, Shim-pack UFLC SHIMADZU CBM30A system^1^; MS, Applied Biosystems 4500 Q TRAP,^2^ Boston, United States) following the methods described by Zou et al. (2020).

### Statistical Analysis

#### One-Way ANOVA

Effects of incubation with different strains were tested using one-way ANOVA. Normality was checked using the Shapiro-Wilk test. The least significant difference (LSD) and Kruskal-Wallis tests were used to identify significant differences for parametric and non-parametric distributions of the estimated parameters, respectively. SPSS 19.0 (IBM, United States) was used for data analysis.

#### MS Data and Statistical Analysis

MS data acquisition and processing were performed following the method described by Chen et al. (2013). Metabolites were annotated using the Metware in-house MS2 spectral tag (MS2T) library (Wuhan Metware Biotechnology Co., Ltd.,^3^ Wuhan, China). Unsupervised principal component analysis (PCA) and supervised multiple regression orthogonal partial least-squares discriminant analysis (OPLS-DA) was conducted using the statistics function prcomp and ropls v1.19.8, respectively, in R (Thevenot et al., 2015). Permutation test (200X) was used to validate supervised models to avoid model overfitting.

In the comparison of metabolites from *Aspergillus niger* and *Bacillus* sp., metabolites with a fold change of ≥ 2 (up-regulated) or ≤ 0.5 (down-regulated) were selected as differentially accumulated metabolites and screened using the threshold variable importance in projection (VIP) value ≥ 1 from the OPLS-DA model to identify differential metabolites. Pathway associations of annotated metabolites were determined by mapping to the Kyoto Encyclopedia of Genes and Genomes (KEGG) pathway database.^4^ The web-based server Metabolite Sets Enrichment Analysis (MSEA)^5^ was used to analyzed pathway enrichment, and pathways with Bonferroni corrected *P*-values of ≤ 0.05 were considered to be significantly enriched.

## Results

### Effects of Different Microbes on Plant Growth and Pb Phytoextraction

The total biomass of *Salix integra* was significantly greater in plants inoculated with all microbial taxa except *Clonostachys rosea*, compared with that of uninoculated controls (*P* < 0.05); notably, biomass was 14% higher under co-inoculation treatment with *Bacillus* sp. and *Aspergillus niger* (Table 2). Inoculation increased Pb concentration in branches and leaves, but not in roots. Pb extraction was promoted by inoculation with *Aspergillus niger* or *Bacillus* sp. alone, and was 10% higher in plants inoculated with *Bacillus* sp. compared with uninoculated control. Furthermore, microbial inoculation mainly promoted accumulation of Pb in aboveground plant parts. The Pb concentration was highest in plants incubated with *Bacillus* sp. in both the branches and leaves. However, inoculation increased transfer coefficients of Pb from root to branch or root to leaf, which were over 100% higher in the co-inoculation treatment compared with those in uninoculated controls.

**TABLE 2:** Effect of inoculation strains on plant growth, lead extraction, and the transfer coefficient.

Index	Organic	CK	*Bacillus* sp.	*Clonostachys rosea*	*Aspergillus niger*	Co-inoculation
Biomass (g)	Root	2.23 ± 0.08a	1.84 ± 0.05c	1.64 ± 0.09c	2.37 ± 0.06b	2.59 ± 0.09a
	Trunk	10.63 ± 0.39b	11.75 ± 0.33b	11.12 ± 0.63b	11.94 ± 0.30b	12.88 ± 0.44a
	Leaf	2.69 ± 0.09c	3.26 ± 0.09a	2.97 ± 0.16b	2.82 ± 0.07b	2.26 ± 0.07d
	Sum	15.55 ± 0.57c	16.85 ± 0.47b	15.73 ± 0.88c	17.13 ± 0.43a	17.73 ± 0.6a
Pb concentration (mg⋅Kg^–1^)	Root	1260.04 ± 46.62a	975.03 ± 27.3b	748.92 ± 41.94c	958.14 ± 23.95b	632.57 ± 21.5d
	Trunk	193.14 ± 7.14d	274.53 ± 7.68a	251 ± 14.05b	222.74 ± 5.57c	217.77 ± 7.40c
	Leaf	160.67 ± 5.94d	253.21 ± 7.09a	173.25 ± 9.70c	218.83 ± 5.47b	185.14 ± 6.29c
Transfer coefficient	Trunk/root	0.15c	0.28b	0.34a	0.23b	0.34a
	Leaf/root	0.13c	0.26a	0.23b	0.23b	0.29a
Amount of enrichment (mg)	Root	2.81 ± 0.10a	1.79 ± 0.05c	1.23 ± 0.07d	2.27 ± 0.06b	1.64 ± 0.06c
	Trunk	2.05 ± 0.07c	3.23 ± 0.09a	2.79 ± 0.15b	2.66 ± 0.07b	2.81 ± 0.10b
	Leaf	0.43 ± 0.02c	0.83 ± 0.02a	0.51 ± 0.03b	0.62 ± 0.02b	0.42 ± 0.01c
	Sum	5.29 ± 0.20c	5.85 ± 0.16a	4.53 ± 0.25d	5.55 ± 0.14b	4.87 ± 0.16d

**Different letters indicate significant differences at p < 0.05. Values represent the mean ± SD (n = 4)*.*

### Effects of Different Microbes on Physiological Index for Plant Stress Tolerance

**FIGURE 2:**
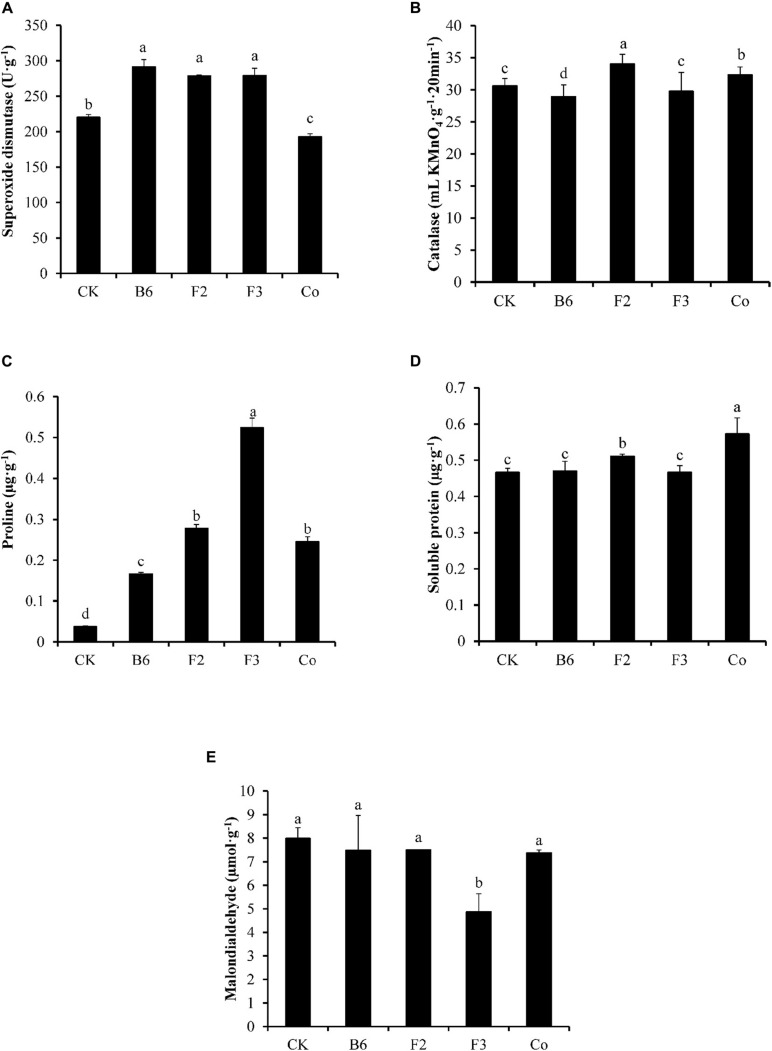
Effects of inoculation different microbes on the physiological index for plant stress tolerance. CK, uninoculated; B6, *Bacillus* sp.; F2, *Clonostachys rosea*; F3, *Aspergillus niger*; Co, Co-inoculation with *Bacillus* sp. and *Aspergillus niger*. **(A)** Superoxide dismutase, **(B)** Catalase, **(C)** Proline, **(D)** Soluble protein, and **(E)** Malondialdehyde. Different letters indicate that the values differed significantly at *p* < 0.05. The values represent the mean ± SD (*n* = 4).

Inoculation of *Salix integra* with different microbial taxa had varying effects on the physiological indices of plant stress tolerance (Figure 2). SOD activity and Pro content were 32% and 3.5 times higher, respectively, in plants treated with *Bacillus* sp. compared with those in untreated controls. Inoculation with *Clonostachys rosea* led to increased activities of SOD and CAT and higher contents of Pro and Sprotein compared with those in untreated controls, with SOD activity and Pro content 27% and 7.39 times those in controls, respectively. Plants inoculated with *Aspergillus niger* exhibited SOD activity and Pro content 27% and 13.98 times higher, respectively, than those in uninoculated controls, while the content of MDA was 38% lower compared with that of controls. Under co-inoculation with *Bacillus* sp. and *Aspergillus niger*, both Pro and Sprotein were higher than those in control plants, with the Pro content being 5.53 times higher than that in the control, while the activities of SOD and CAT were lower.

### Effects of Microbial Inoculation on Antioxidant Enzyme Activity of Soil

**FIGURE 3:**
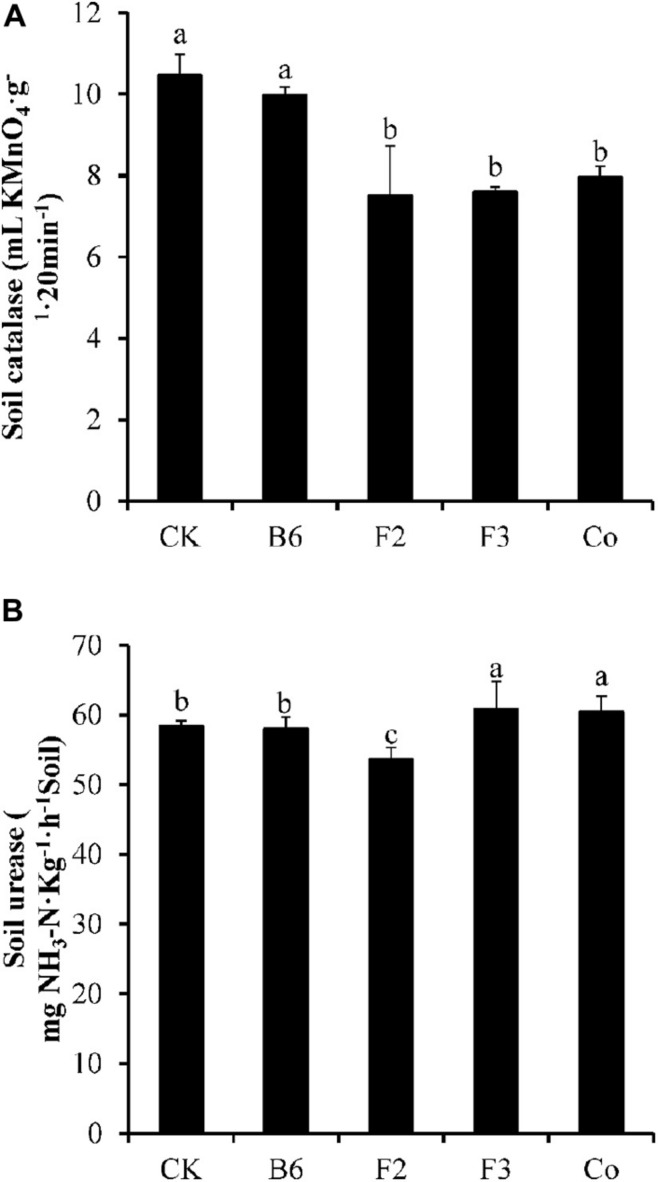
Effects of microbial inoculation on antioxidant enzyme activity of the soil. CK, uninoculated; B6, *Bacillus* sp.; F2, *Clonostachys rosea*; F3, *Aspergillus niger*; Co, Co-inoculation with *Bacillus* sp. and *Aspergillus niger*. **(A)** Soil catalase and **(B)** soil urease. Different letters indicate that the values significantly differed at *p* < 0.05. The values represent the mean ± SD (*n* = 4).

Inoculation of *Salix integra* with different microbial taxa had only small effects on the antioxidant enzyme activity of the soil (Figure 3). Inoculation with *Bacillus* sp. did not significantly affect the activities of S-CAT or S-UE; however, soil enzyme activities were lower in soil of plants inoculated with *Clonostachys rosea* than in that of uninoculated plants. S-CAT activity was lower and S-UE activity was higher compared with controls in soil of plants inoculated with *Aspergillus niger* or co-inoculated with *Bacillus* sp. and *Aspergillus niger*.

### Effects of Microbial Inoculation on Heavy Metal Speciation in Rhizosphere and Bulk Soil

**FIGURE 4:**
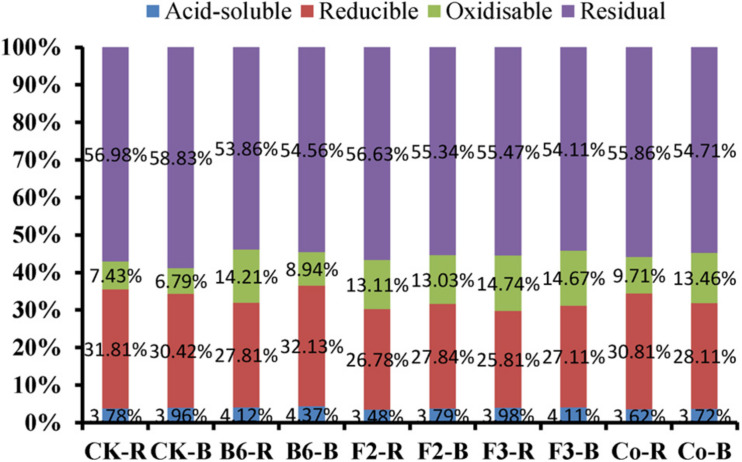
Effects of microbial inoculation on heavy metal speciation in the rhizosphere and bulk soil. CK-R, rhizosphere soil in CK; CK-B, bulk soil in CK; B6-R, rhizosphere soil in inoculation with *Bacillus* sp.; B6-B, bulk soil in inoculation with *Bacillus* sp.; F2-R, rhizosphere soil in inoculation with *Clonostachys rosea*; F2-B, bulk soil in inoculation with *Clonostachys rosea*; F3-R, rhizosphere soil in inoculation with *Aspergillus niger*; F3-B, bulk soil in inoculation with *Aspergillus niger*; Co-R, rhizosphere soil in inoculation with *Bacillus* sp. and *Aspergillus niger*; Co-B, bulk soil in inoculation with *Bacillus* sp. and *Aspergillus niger*.

The ratios of Pb species in the four soil fractions were relatively consistent under different treatments, with levels of residual and reducible Pb higher than those of oxidizable and acid-soluble Pb (Figure 4). Microbial inoculation changed the speciation of heavy metals in the rhizosphere and bulk soil of *Salix integra*. Acid-soluble Pb proportions in rhizosphere and bulk soil were 0.34 and 0.41% higher under inoculation with *Bacillus* sp. than those in the control treatment, with *Aspergillus niger* inoculation showing a similar but lesser effect. However, residual Pb proportions were all lower in soils of inoculated plants than in those of controls, and were lowest in the rhizosphere soil under inoculation with *Bacillus* sp. Additionally, the proportion of acid-soluble Pb was lower in rhizosphere soil than in bulk soil, which may contribute to absorption by roots.

### Widely Targeted Metabolome Analysis of Microbes

*Aspergillus niger* and *Bacillus* sp. showed the largest promotion effect on Pb absorption of *Salix interga*. This effect was conferred by metabolites produced by these microorganisms after inoculation, so we conducted widely targeted LC-MS/MS-based metabolite profiling of these microbes. We identified a total of 410 metabolites, more than 50% of which were likely to contribute to the bioavailability of heavy metals, including 82 amino acids, 89 organic acids, 31 carbohydrates, and 32 nucleotides, as well as other primary and secondary metabolites (Supplementary Table 1).

#### Metabolite Profile Analysis of *Aspergillus niger* and *Bacillus* sp.

We conducted PCA analysis on the 410 metabolites, which were distinctly divided into three groups associated with *Aspergillus niger*, *Bacillus* sp., and the quality control (QC), respectively (Figure 1A). The peak area of each metabolite was transformed by log10 to alleviate the effects of quantity on pattern recognition, and then metabolites were analyzed using hierarchical cluster analysis. The metabolites formed two distinct groups relative to *Aspergillus niger* and *Bacillus* sp. (Figure 1B), implying that the metabolite profiles of these two microorganisms were obviously different.

**FIGURE 5:**
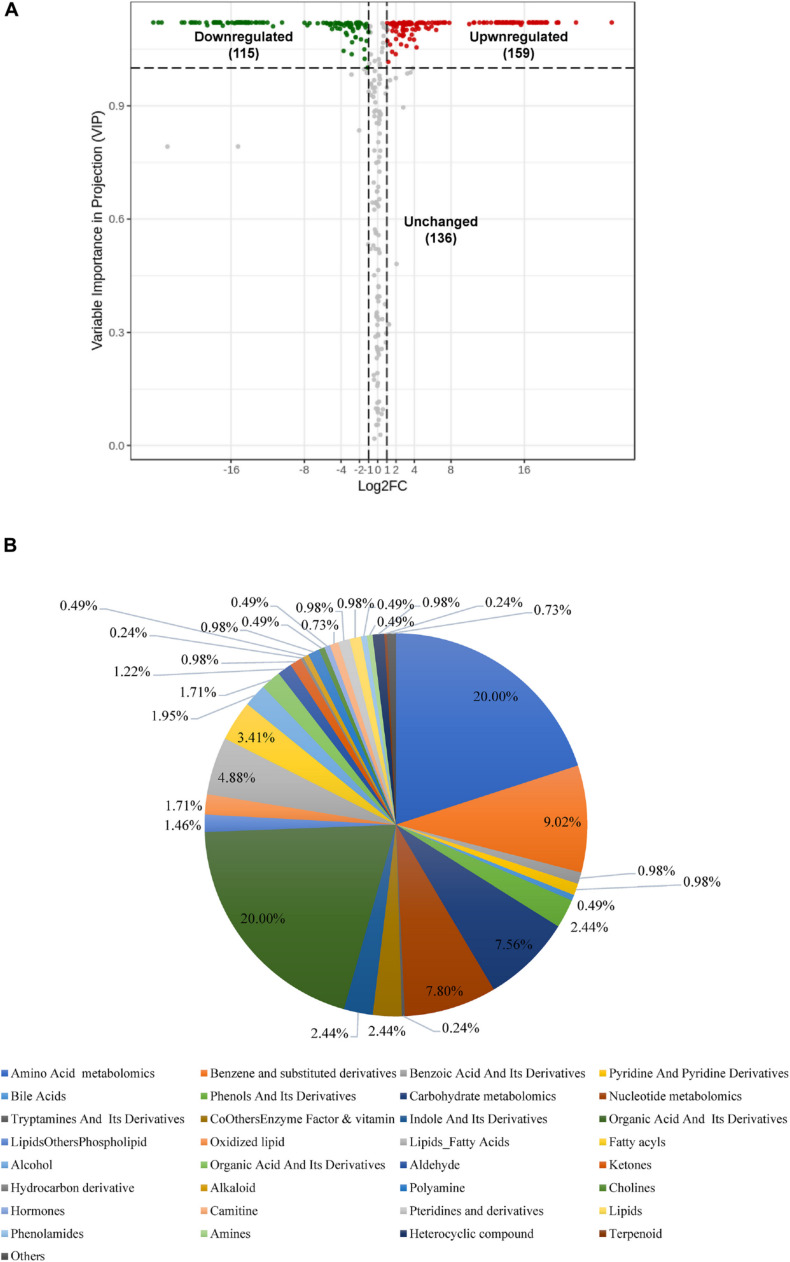
Differential accumulation of metabolites between *Aspergillus niger* and *Bacillus* sp. **(A)** Volcano plot of the 410 identified metabolites. Differential metabolites were defined as metabolites with a fold change of ≥ 2 or ≤ 0. 5 in *Aspergillus niger* compared to *Bacillus* sp. A threshold of VIP ≥ 1 was used to separate differential metabolites from unchanged metabolites. **(B)** Pie chart depicting the biochemical categories of the differential metabolites identified between *Aspergillus niger* and *Bacillus* sp.

When comparing *Bacillus* sp. and *Aspergillus niger*, we identified 274 metabolites as differentially produced between species (Figure 5A and Supplementary Table 2). Of these, 115 metabolites were down-regulated and 159 were up-regulated in *Bacillus* sp. compared with *Aspergillus niger* (Figure 5A). The 274 metabolites could be categorized into more than 18 different classes (Figure 5B); however, most of them were organic acids, amino acids, or carbohydrates, and their derivatives (Supplementary Table 2).

#### KEGG Classification and Enrichment Analysis of Differential Metabolites

The 274 metabolites showing differential production between *Aspergillus niger* and *Bacillus* sp. were mapped to the KEGG database to determine which metabolic pathways they might be associated with. Most of the metabolites were mapped to metabolism (Supplementary Figure 1), as expected. Some were categorized as organismal system or human diseases, implying that they may have effects on health. A few were classified as cellular process, environmental information processing, drug development, or genetic information processing (Supplementary Figures 2, 3). We conducted KEGG pathway enrichment analysis to identify differences in the metabolic pathways between the two microbes, which revealed significant differences (*p* < 0.05) in metabolic pathways between the two microorganisms, particularly in histidine, tyrosine, and tryptophan metabolism (Supplementary Figure 4).

#### Effects of Metabolites to Heavy-Metal Bioavailability and Plant Growth

We were focused on metabolites having important effects on heavy-metal bioavailability, such as amino acids, organic acids, and carbohydrates. Organic acids can increase, decrease, and have no effect on Pb bioavailability depending on environmental factors such as pH or water. However, amino acids and carbohydrates can combine with Pb^2+^, which might decrease Pb bioavailability and toxicity.

We identified 78 and 81 organic acids produced by *Aspergillus niger* and *Bacillus* sp. (Supplementary Table 1), respectively, some of which exhibited high relative abundances, such as aspirin, L-dihydroorotic acid, succinic anhydride, proline betaine, and 6-aminocaproic acid in *Aspergillus niger*, and proline betaine, methylmalonic acid, 6-aminocaproic acid, trans-3-Indoleacrylic acid, and cinnamic acid in *Bacillus* sp. Sixty organic acids were differentially produced between the two microbes, and all exhibited significant differences in quantities (fold change of ≥ 2 or ≤ 0.5, and VIP ≥ 1; Supplementary Table 2). Thirty-seven of these organic acids were up-regulated, while 23 organic acids were down-regulated in *Bacillus* sp. compared with *Aspergillus niger*.

There were 65 and 75 amino acids produced by *Aspergillus niger* and *Bacillus* sp. (Supplementary Table 1), respectively. The relative abundance of some metabolites produced by *Aspergillus niger* was high, such as L-glutamic acid, L-valine, DL-leucine, L-proline, L-tyrosine, and L-asparagine anhydrous, meanwhile *Bacillus* sp. produced high quantities of L-leucine, N-acetylglycine, L-tyrosine, L-valine, Trans-4-hydroxy-L-proline, and DL-leucine. Sixty-nine amino acids were differentially produced, and their quantities exhibited significant differences (fold change ≥ 2 or ≤ 0.5, and VIP ≥ 1). Of these, the relative abundance of 44 amino acids was up-regulated and 25 amino acids were down-regulated in *Bacillus* sp. compared with *Aspergillus niger*.

Twenty-eight and twenty-three carbohydrates were produced by *Aspergillus niger* and *Bacillus* sp. (Supplementary Table 1), respectively. The relative abundances of D-fructose, D-mannose, and D-glucose were higher in the culture supernatant of *Aspergillus niger*, while those of 6-phosphogluconic acid trisodium salt, D-mannose, and D-erythronolactone were higher in the culture supernatant of *Bacillus* sp. Based on fold-changes and VIP values, we identified 28 carbohydrates as differentially produced between the two microbes; of these, the relative abundances of six carbohydrates were up-regulated and those of 22 carbohydrates were down-regulated in *Bacillus* sp. compared with *Aspergillus niger*.

Some metabolites can promote plant growth, such as indole-3-acetic acid, 1-naphthylacetic acid, and adenine. These were found in both microbes, although their relative abundances were lower than those of amino acids, organic acids, and carbohydrates (Supplementary Table 2).

## Discussion

Heavy-metal-resistant microbes with plant-growth-promoting traits have been used as bioinoculants to increase plant biomass and accumulation of heavy metals (Ijaz et al., 2016; Ma et al., 2017; Fan et al., 2018; Wang L. et al., 2018). However, their application still has some problems. The effects of inoculation with indigenous metal-resistant microbes on phytoremediation have often been overlooked. Therefore, we conducted a greenhouse experiment using *Salix integra* growing in Pb-contaminated soil to investigate the effects of inoculation with indigenous metal-resistant microbes on phytoremediation and examine the mechanism of microbial promotion of the phytoremediation process.

### Single and Co-inoculation Have Different Effects on Growth and Pb Accumulation of *S. integra*

Plant growth and Pb transfer coefficients were both enhanced by inoculation with *Bacillus* sp., *Aspergillus niger*, and their co-inoculation. However, Pb accumulation was increased by single inoculation, but not by co-inoculation, which may be attributed to interactions between the two microorganisms (Gola et al., 2016; Sarathambal et al., 2017). Previous studies have reported that co-inoculation effects are very complicated (Tsimilli-Michael et al., 2000; Yu et al., 2005; Aghababaei et al., 2014; Mahohi and Raiesi, 2019). Inoculation treatment mainly promoted the accumulation of Pb in aboveground plant parts, consistent with increases in transfer coefficients. Only the transfer coefficients were increased after inoculation with *Clonostachys rosea*. From these results, we infer that inoculated microbes play different roles in phytoremediation and their interactions should be considered in the application of co-inoculation (Chen et al., 2019).

### Microbes Affect Physiological Metabolites and Enhance Plants Tolerance to Heavy Metals

The activities of SOD or CAT in *Salix integra* were enhanced by inoculation with microbes. Generation of reactive oxygen species (ROS), such as superoxide free radicals (O^2–^), hydroxyl free radicals (OH^–^), and hydrogen peroxide (H_2_O_2_), is increased in the presence of excessive heavy metals through Fenton-like reactions in plant cells, harming the plant (Tsang et al., 1996). The antioxidase system removes ROS through sequential and simultaneous enzymatic catalysis reactions (Wu et al., 2020c). Therefore, increases in the activities of SOD and CAT suggest that the *Salix integra* antioxidant defenses were promoted after inoculation with microorganisms (Becana et al., 2000; Kong et al., 2015; Hidri et al., 2019; Ju et al., 2020). MDA (a cytotoxic product of lipid peroxidation) level is believed to be the best measure of lipid peroxidation status and cell membrane damage induced by ROS production (Li et al., 2013). We observed no significant changes in MDA between the different inoculation treatments and uninoculated controls, except inoculation with *Aspergillus niger* caused a significant decrease in the MDA content of leaves. This suggests that Pb enrichment is not harmful to inoculated *Salix integra* (Huang et al., 2020), proving again that *Salix integra* antioxidant defenses are promoted after microbial inoculation.

Both Pro and Sproteins can combine with available metal ions in plant cells, thereby alleviating their cytotoxicity (Xin et al., 2020). We recorded significantly higher Pro accumulation in leaves under all inoculation treatments compared with that in uninoculated controls, possibly due to the increased Pb enrichment in plants after inoculation. Plants accumulate free amino acids, particularly Pro, to alleviate harm under stressful conditions. These are crucial mechanisms for the adaptation of *Salix integra* to Pb stresses (Ghoulam et al., 2002; Farhangi-Abriz and Torabian, 2017; Xin et al., 2020). The content of Sproteins was only higher compared with uninoculated controls under inoculation with *Clonostachys rosea* or co-inoculation with *Bacillus* sp. and *Aspergillus niger*, which is consistent with the higher transfer coefficients in these treatments. However, in previous work, Pro content was reduced under heavy-metal conditions in the roots of plants inoculated with arbuscular mycorrhizal fungi, which reduced plants’ exposure to metals by sequestering them into their own structures (Kanwal et al., 2015; Akhtar et al., 2020; Huang et al., 2020). Therefore, the mechanisms of plant tolerance to heavy metals imparted by inoculation with microorganisms may be diverse. Our results imply that specific microbes affect plant metabolites, enhancing resistance to heavy metals.

### Microbial Inoculation Enhances Pb Bioavailability

The speciation and bioavailability of heavy metals in soils are principal factors affecting the efficiency of phytoremediation. Microbial inoculation significantly accelerated the conversion of residual Pb to the other three fractions in the rhizosphere and bulk soil of all treatments. Studies have shown that microorganisms can alter the mobility of heavy metals and their availability for plants (Huang et al., 2020). The proportion of acid-soluble Pb was higher than that in controls only after inoculation with *Bacillus* sp. or *Aspergillus niger*, but not in the other treatments. Acid-soluble Pb is the speciation that plants can absorb directly. Therefore, a higher proportion of acid-soluble Pb might promote Pb accumulation by *Salix integra*, which is consistent with the greater enrichment of Pb in these two treatments. Therefore, Pb bioavailability is promoted by inoculation of soil with *Bacillus* sp. and *Aspergillus niger*.

Soil enzymes play fundamental roles in the regulation of biochemical transformation. CAT is a type of oxidoreductase related to the activity of aerobic microorganisms (Kang et al., 2018), and urease plays an important role in the nitrogen cycle in soils (Hu et al., 2014). In this study, only the activity of S-UE was enhanced by inoculation with *Clonostachys rosea* or co-inoculation with *Bacillus* sp. and *Aspergillus niger*. A higher content of acid-soluble Pb decreases soil enzyme activity, which was consistent with the higher proportion of acid-soluble Pb in these two treatments (Yu et al., 2019). Therefore, microbial inoculation had little positive effect on soil biochemical properties.

From the above results, we imply that specific microorganisms colonize the soil and enhance Pb bioavailability to assist phytoremediation, but they have little positive effect on soil biochemical properties.

### Widely Targeted Metabolome Analysis of Microbes

Widely targeted metabolite profiling analysis utilizing MS/MS data has previously been used successfully for large-scale metabolite profiling and comparative metabolomics of several important plant species (Oikawa et al., 2015; Wang et al., 2016, 2017; Wang D.D. et al., 2018). However, its application is limited in microbial metabolomics, and previous studies on the metabolites of microbes used for plant inoculation have focused on specific classes of metabolites, such as indole-3-acetic acid (Fatima and Ahmed, 2018; Rehman et al., 2019), chelating compounds, organic acids (Ashraf et al., 2018; Biswas et al., 2018; Rehman et al., 2019), amino acids, biosurfactants (Yang et al., 2016), and siderophores. Over-arching metabolic profiles of such microorganisms have not been thoroughly investigated until now.

We used LC-MS/MS-based widely targeted metabolomics to understand the metabolic profiles of two Pb-resistant microorganisms and the mechanism by which they assist phytoremediation. We identified 410 metabolites, of which over 50% of them contribute to the bioavailability of heavy metals, such as amino acids, organic acids, carbohydrates, nucleotides, and other primary and secondary metabolites (Luo et al., 2017). Therefore, this study provides novel evidence that microbial metabolites have significant effects in activating heavy metals and promoting phytoremediation (Yang et al., 2016; Jian et al., 2019; Akhtar et al., 2020; Huang et al., 2020; Ju et al., 2020; Tao et al., 2020).

*Bacillus* sp. and *Aspergillus niger* cultures exhibited distinct metabolite profiles, and their metabolic pathways were significantly different, particularly for amino acids. There were 274 significantly differentially produced metabolites between the two taxa, and most of these were organic acids, amino acids, carbohydrates, and their derivatives. This implies that the different effects observed by inoculation with different microbes are mainly attributed to organic acids, amino acids, and carbohydrates. Therefore, we should focus on these three categories of metabolites to obtain a clearer understanding of the mechanism involved in promoting phytoremediation.

Of the 410 metabolites, the ratio of organic acids was highest, followed by amino acids, which might change the chemical speciation of heavy metals in the two microorganisms (Luo et al., 2017). We also identified growth-promoting substances among the metabolites, such as indole-3-acetic acid, 1-naphthylacetic acid, and adenine; however, their abundances were lower than those of organic and amino acids. We therefore speculate that *Bacillus* sp. and *Aspergillus niger* mainly assist phytoremediation by changing the speciation of heavy metals.

In our study, the microbial taxon used for inoculation not only affected Pb accumulation but also affected transfer coefficients, implying that the inoculated microbes colonized the soil and plant at the same time and their metabolite patterns are different under different living conditions. In soil, the metabolites of *Bacillus* sp. and *Aspergillus niger* mainly activated heavy metals, while they mainly chelated heavy metals in the plant. This deserves further study. Additionally, the effects of co-inoculation were different from those of single inoculation, and we speculate that the metabolic patterns of the two microorganisms were changed by mutual effects. It would be interesting to measure the over-arching metabolic profile of the co-inoculation treatment.

## Conclusion

Single inoculation of *Salix integra* with *Bacillus* sp. or *Aspergillus niger* promotes Pb absorption and the accumulation of Pb in aboveground plant parts. *Bacillus* sp. and *Aspergillus niger* mainly assist phytoremediation by changing the speciation of heavy metals and enhancing the tolerance of *Salix integra* to heavy metals. Differences in the metabolite profiles and metabolic pathways of the two microorganisms are mainly in organic acids, amino acids, and carbohydrates; therefore, future studies should focus on these three categories of metabolites to obtain a clearer understanding of the mechanism involved in promoting phytoremediation. We speculate that inoculated microbes colonize the soil and plant at the same time and their metabolite patterns are different under different living conditions, although this requires further study. Additionally, co-inoculation with *Bacillus* sp. and *Aspergillus niger* had different effects from single inoculation on Pb accumulation; however, the mechanism of these differences remains unclear and requires further study. We also need to consider interactions between inocula and the whole microbial community when applying microbial inoculation to promote phytoremediation.

## Data Availability Statement

The original contributions presented in the study are included in the article/Supplementary Material, further inquiries can be directed to the corresponding author/s.

## Author Contributions

XN: conceptualization, investigation, data analysis, and writing—original draft. SW: investigation, methodology, laboratory experiment, and writing—original draft. DH: supervision, project administration, funding acquisition, and writing—review and editing. JZ: field experiment and data analysis. DD and PS: laboratory experiment and data analysis. All authors provided critical feedback, helped to shape the research, analysis, and the manuscript. All authors discussed the results and contributed to the final version of the manuscript.

## Conflict of Interest

The authors declare that the research was conducted in the absence of any commercial or financial relationships that could be construed as a potential conflict of interest.

## Publisher’s Note

All claims expressed in this article are solely those of the authors and do not necessarily represent those of their affiliated organizations, or those of the publisher, the editors and the reviewers. Any product that may be evaluated in this article, or claim that may be made by its manufacturer, is not guaranteed or endorsed by the publisher.

## Footnotes

1
www.shimadzu.com.cn

2
www.appliedbiosystems.com.cn

3
http://www.metware.cn

4
http://www.kegg.jp/kegg/pathway.html

5
http://www.msea.ca
