# Supplementary figures and images for: Inoculation With Indigenous Rhizosphere Microbes Enhances Aboveground Accumulation of Lead in Salix integra Thunb. by Improving Transport Coefficients
Source: Front Microbiol. 2021 Aug 4;12:686812. doi: 10.3389/fmicb.2021.686812 (PMC8371752; doi:10.3389/fmicb.2021.686812)

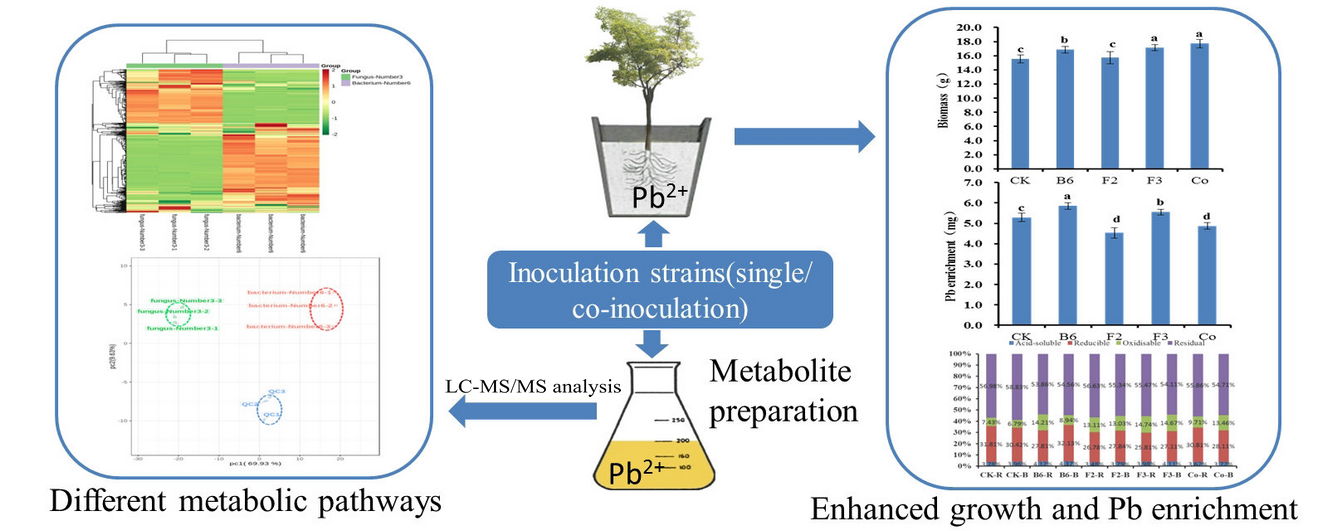

Supplement: Supplementary file 1 [file Image_1.TIF]
