# Supplementary material for: Inoculation With Indigenous Rhizosphere Microbes Enhances Aboveground Accumulation of Lead in Salix integra Thunb. by Improving Transport Coefficients
Source: Front Microbiol. 2021 Aug 4;12:686812. doi: 10.3389/fmicb.2021.686812 (PMC8371752; doi:10.3389/fmicb.2021.686812)
Supplement: Supplementary file 2 [file Data_Sheet_1.docx]

Supplementary Material

# Supplementary Figures

| **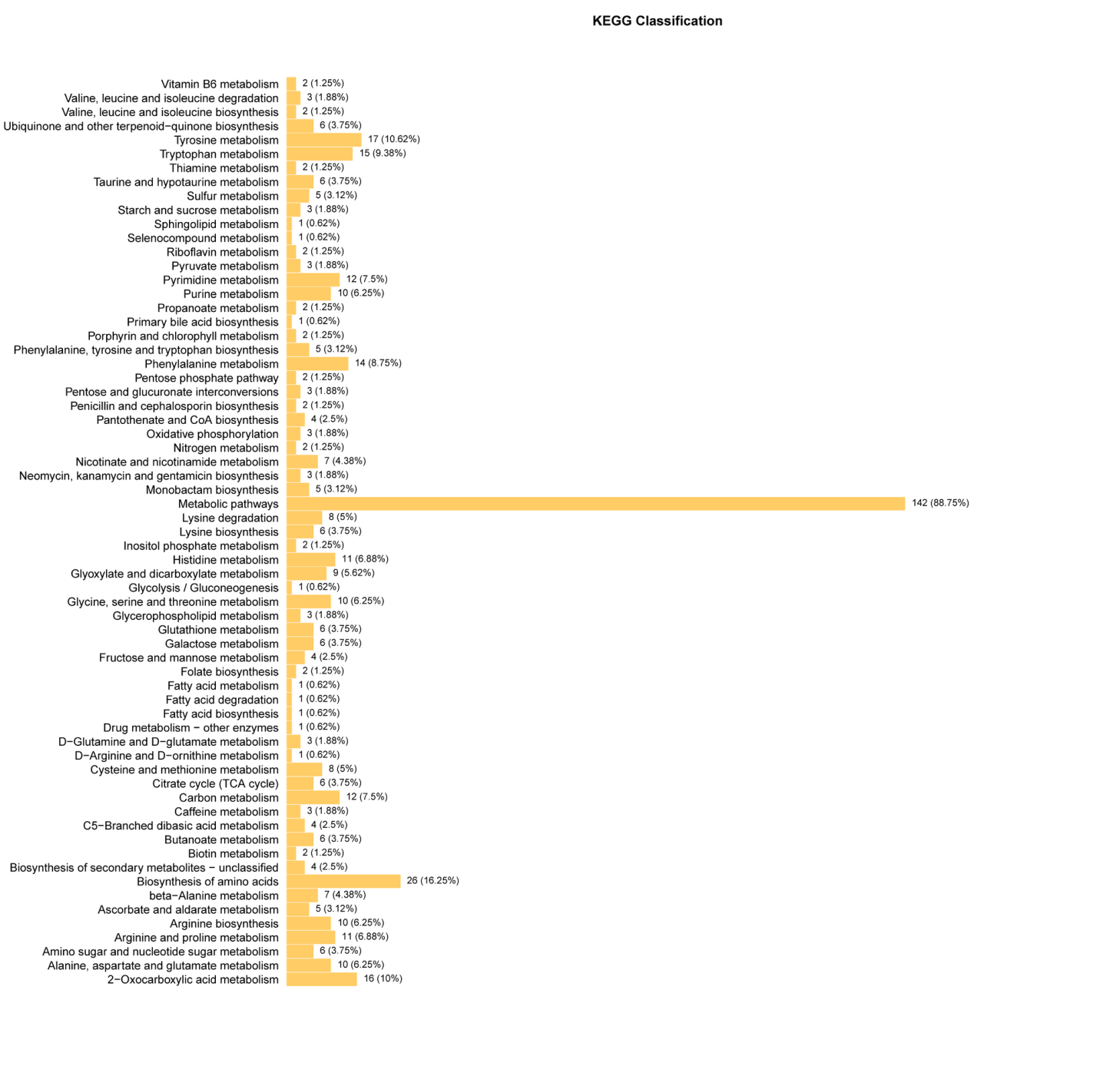** |
| --- |
| **Fig. S1. KEGG classification of differentially accumulating metabolites (system** **information: Metabolism).** Most of the identified metabolites were classified as “Metabolism” in the KEGG database. |

| 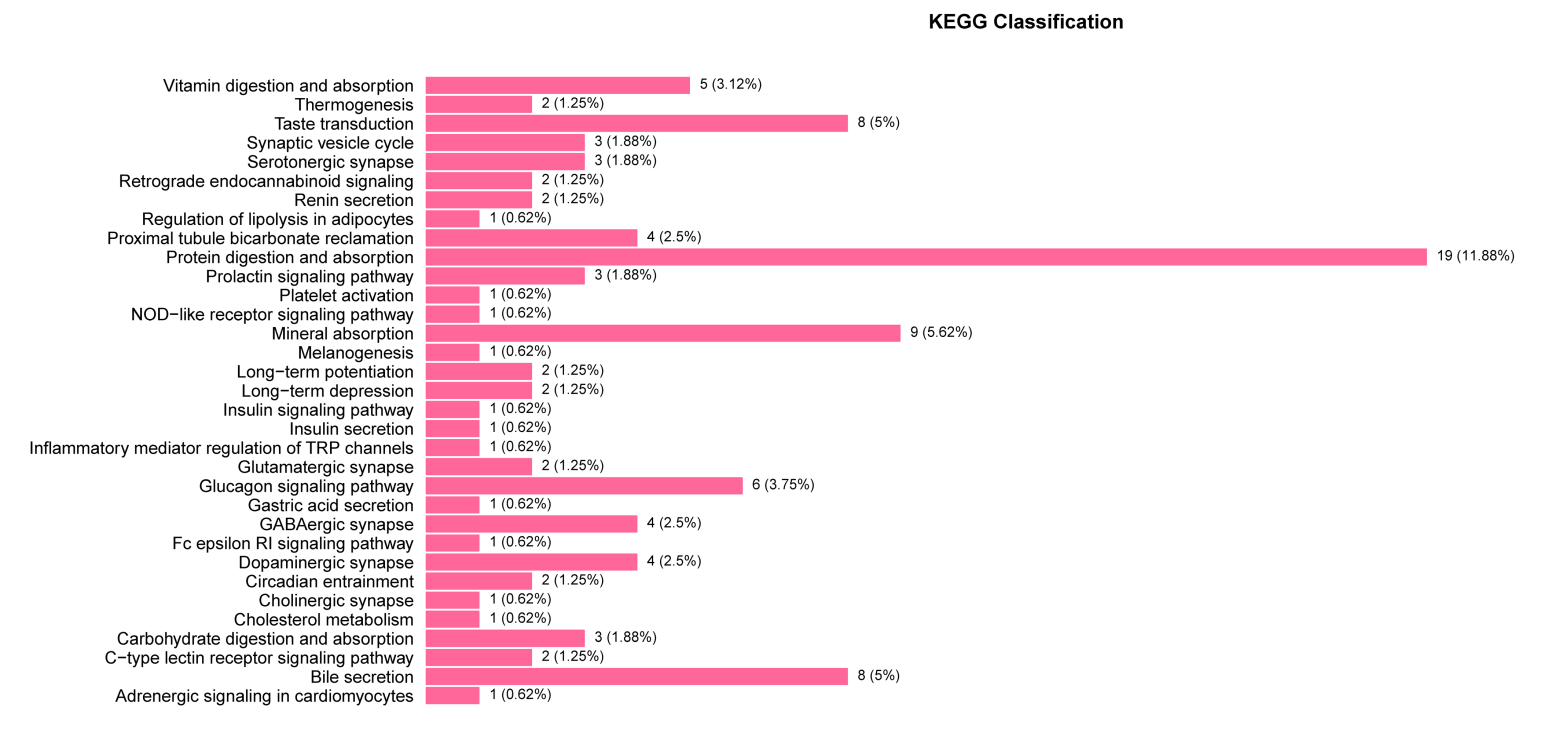 |
| --- |
| **Fig. S2. KEGG classification of differentially accumulating metabolites (system** **information: Organismal system).** Number of metabolites associated with “Organismal system” is shown. |

| 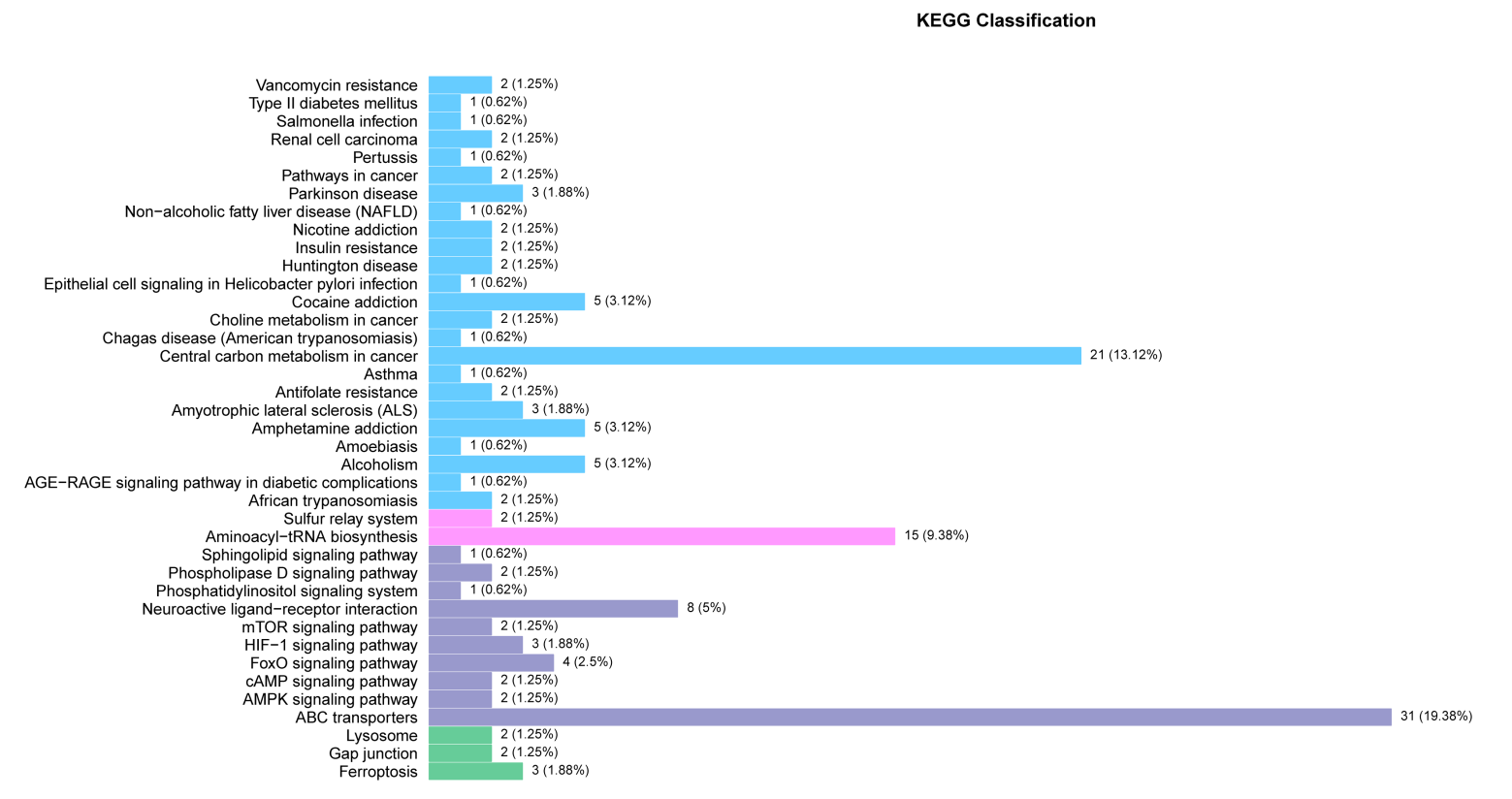 |
| --- |
| **Fig. S3. KEGG classification of differentially accumulating metabolites (Other classes of** **system information).** Number of metabolites associated with other system information is shown. |

| 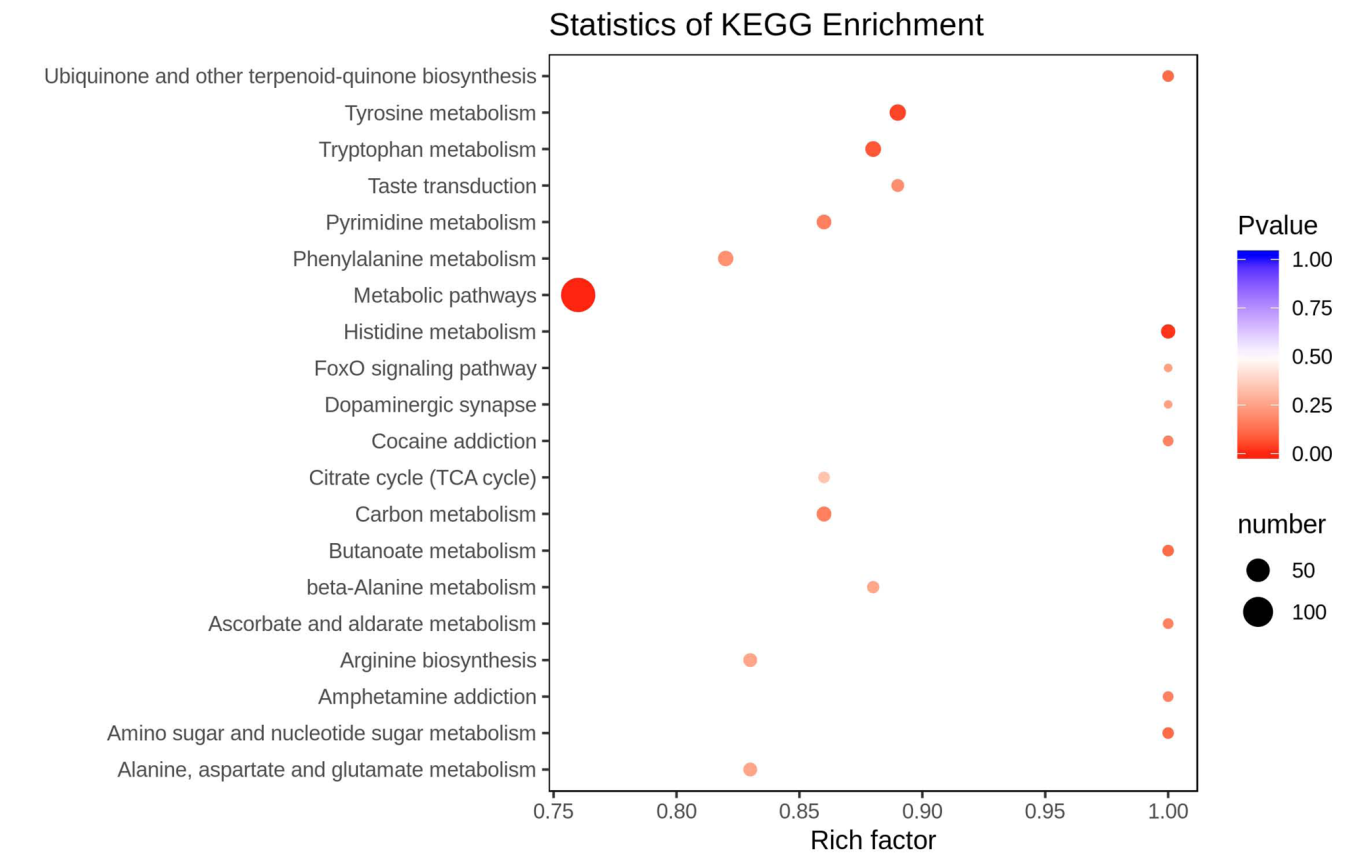 |
| --- |
| **Fig. S4. KEGG enrichment analysis of differentially accumulating metabolites between** ***Bacillus* sp. and *Aspergillus niger*.** Each circle in the plot represents the number of associated metabolites and is position according to its rich factor. The *p*-values are indicated by colors. |
